# Polysaccharides from the Edible Mushroom *Agaricus bitorquis* (Quél.) Sacc. Chaidam Show Anti-hypoxia Activities in Pulmonary Artery Smooth Muscle Cells

**DOI:** 10.3390/ijms20030637

**Published:** 2019-02-01

**Authors:** Yingchun Jiao, Hui Kuang, Jianan Wu, Qihe Chen

**Affiliations:** 1 Agriculture and Animal Husbandry College, Qinghai University, Xining 810016, China; jiaoyingchun@qhu.edu.cn; 2 Department of Food Science and Nutrition, Zhejiang University, Hangzhou 310058, China; 11813032@zju.edu.cn (H.K.); 21613036@zju.edu.cn (J.W.)

**Keywords:** *Agaricus bitorquis* (Quél.) Sacc. Chaidam, intracellular polysaccharides, structural characterization, anti-hypoxia activity

## Abstract

Three kinds of new water-soluble polysaccharides (FA, FB and FC) were isolated from wild mushroom *Agaricus bitorquis* (Quél.) Sacc. Chaidam by the classical method “water extraction and alcohol precipitation” and purified by column chromatography. The Mw of FA, FB and FC ranged from 5690 Da to 38,340 Da. The three polysaccharide fractions in the fruiting body were mainly composed of 4 kinds of monosaccharides, including glucose, galactose, mannose, and arabinose, among which glucose and galactose were the major monosaccharides. The FTIR and NMR spectroscopy indicated that the skeleton of three fractions composed of a (1→4)-α-D-glycosidic backbone containing α-D-mannopyranose. In vitro anti-hypoxia activity data showed that three polysaccharide fractions possessed a significant effect on inhibiting PASM cells apoptosis under hypoxia. Among them, FC at the concentration of 200 µg/mL revealed a significant anti-hypoxia effect. These results revealed that the intracellular polysaccharides possessed potent anti-hypoxic activity, which might be related to inhibiting LDH and NADPH oxidase expression and promoting the formation of 5-hydroxytryptamine, dopamine, endothelins, acetylcholine. More importantly, FC showed good performance inducing KV1.5 expression and prohibiting KIR6.2 formation at protein level.

## 1. Introduction

Oxygen is essential for mammalian metabolism and physiological functions because of its use in cellular energy production and cofactor/substrate for many enzymes [1]. Under hypoxia conditions, oxygen supply in various tissues of the body decreases. Furthermore, hypoxia induces the generation of lipid peroxidation and oxygen free radicals in mammalian tissues [2,3]. Hypoxia-inducible factor (HIF), a central regulator for detecting and adapting to cellular oxygen levels, transcriptionally activates genes modulating oxygen homeostasis and metabolic activation. Beyond this, HIF influences many other processes [1]. The upregulation of HIF is related to a number of gene hallmarks of cancer, including cell proliferation, metabolic reprogramming, apoptosis, invasion and metastasis, and resistance to therapies [4]. Anti-hypoxia medicines like dexamethasone, amphetamine and propranolol have exhibited protective and relieving effects against the damage of hypoxia on human. However, in spite of their curative activity, their application was limited because of the side effects these medicines possess. Rhodiolaalgida, also named Hongjingtian in China, is the most famous Tibetan medicinal plant used to inhibit acute mountain sickness (AMS) [5]. Though some traditional Chinese herb extractions including rhodiola extracts, ginkgolbiloba extracts, ginseng extracts, and some traditional Chinese medicines, exhibit anti-hypoxia effects, their development and application were still discouraged by the shortage of these resources, their expensive prices and the limited distribution of their geographical locations [6]. Thus, it is vital and urgent for scientists to find anti-hypoxia bioactive compounds which are easily available, natural, nontoxic and effective for hypoxia prevention and curation.

Hypoxia pulmonary arterial hypertension (HPAH) is a common pathological processes of mountain sicknesses, chronic pulmonary heart disease and pulmonary edema [7,8]. Pulmonary artery smooth muscle cell (PASMC) plays an important role in HPAH. Hypoxia-induced PASMC apoptosis results in pulmonary vascular remodeling which is the pathological basis of HPAH [9]. Thus, in the present study, PASMC was selected as the cell model to evaluate the anti-hypoxia effects.

Previously, mushrooms have been regarded as a low calorie, low fat option with abundant beneficial nutrition. Edible mushroom is considered to be a valuable source of dietary ingredients necessary for stimulating the development and growth of human organism and sustaining life functions [10]. Besides, mushrooms which are rich in polysaccharides [11,12] and phenolic compounds [13] are considered to be harmless sources of natural antioxidants since those two compounds are concerned with the antioxidant activity of fungi. However, in the past decade, much research has demonstrated that mushroom is a nutritious diet component which is capable of preventing and treating chronic diseases [14]. In our search for promising anti-hypoxic food sources, we found *Agaricus bitorquis* (Quél.) Sacc mushroom exhibited significant neuroprotective properties in preliminary studies. *A. bitorquis* (Quél.) Sacc. is a wild large edible fungus growing on the Chaidam basin in Tibet Plateau. It is mainly distributed in Delingha, Nomuhong, Golmud and other districts of Chaidam basin at the altitude of 2600 m-3000 m. This mushroom has the unique characteristics of a big fruiting body, strong stress resistance, solid root and adaptation to low temperature. Having ample nutrients, it is heavily utilized by local herdsmen as an anti-hypoxic and anti-fatigue ingredient for consumption and selling. However, limited literatures were conducted with regard to the anti-hypoxia elucidation of polysaccharides from fruiting body of *A. bitorquis* (Quél.) Sacc. Chaidam. The present study, therefore, aimed to examine the effect of intracellular polysaccharides extracted from the fruiting body on hypoxia-induced PASMC so as to elucidate the anti-hypoxia activity and possible underlying mechanism, as well as to provide scientific evidence for the use of such a special mushroom variety.

## 2. Results and Discussion

### 2.1. Partial Purification and Structural Characterization of Polysaccharides

**Figure 1:**
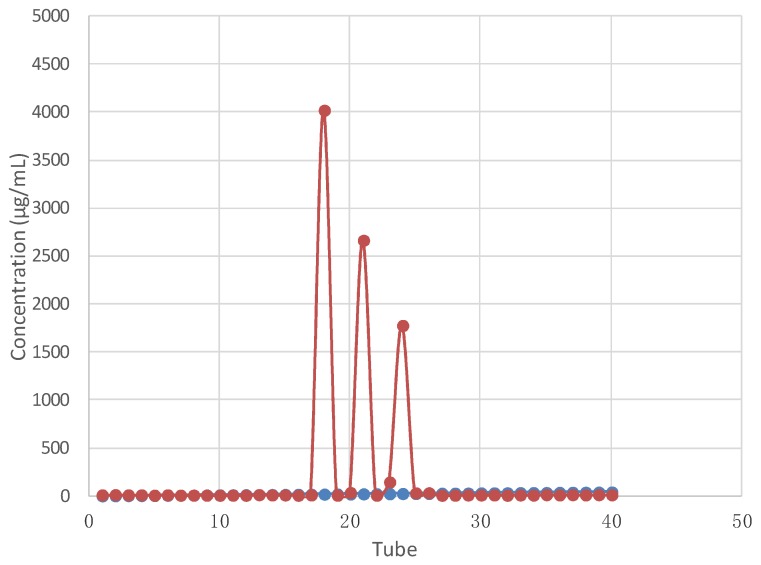
Elution curve of three polysaccharide fractions purification with HPGPC chromatograms.

Intracellualr polysaccharides (IPSs) obtained from the fruiting body of A. bitorquis (Quél.) Sacc. Chaidam were fractionated by DEAE-52 and Sephacryl S-200 size-exclusion chromatography to obtain three purified fractions, named FA, FB and FC, respectively (Figure 1), which were selected on the basis of the elution curves. The molecular weights (MW) of FA, FB and FC were estimated by the SEC-MALLS-RI system (figures not shown). According to the dextran standards, the average molecular mass of FA, FB and FC was estimated to be 38,340 Da, 12,600 Da, 5690 Da, respectively.

**Figure 2:**
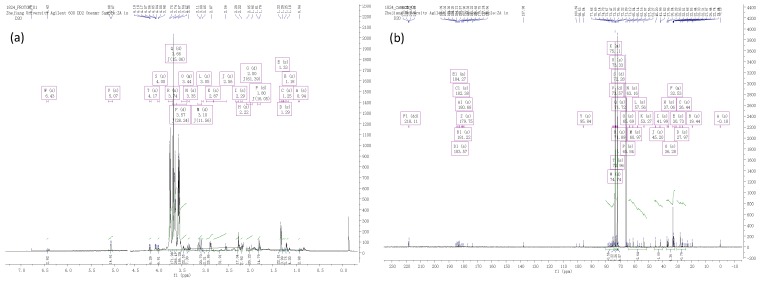

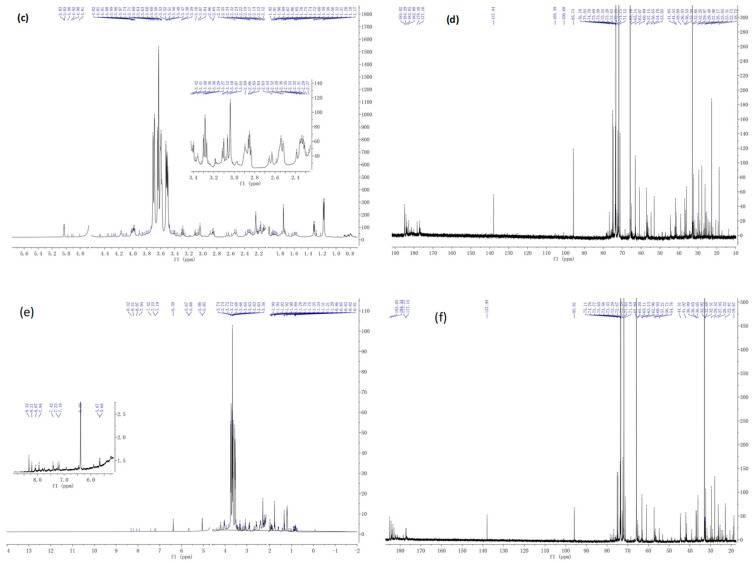
NMR spectra of three polysaccharides fractions. (**a**) ^1^H NMR spectrum of FA; (**b**) ^13^C NMR spectrum of FA; (**c**) ^1^H NMR spectrum of FB; (**d**) ^13^C NMR spectrum of FB; (**e**) ^1^H NMR spectrum of FC; (**f**) ^13^C NMR spectrum of FC.

A PMP-HPLC method was used to analyze the monosaccharides composition of the EPS fractions. The intracellular polysaccharides in *A. bitorquis* (Quél.) Sacc. Chaidam fruiting body were mainly composed of four monosaccharides: glucose, galactose, mannose, and arabinose. Other sugars such as rhamnose, galacturonic acid or glucuronic acid were not detected in the hydrolyzed products (data not shown). Glucose and galactose were the major monosaccharides. The molar ratio of monosaccharide compositions of glucose, mannose, rhamnose, and galactose in FA was described as 1.00:0.26:0.62:0.80. In FB, the molar ratio of glucose, mannose, and galactose was 1.00:0.40:0.54, with glucose as the major monosaccharide. In FC fraction, the molar ratio of glucose, mannose, and galactose was 0.80:0.70:0.65. Based on the signals of FA, FB and FC in ^1^H and ^13^C NMR spectra (Figure 2), the primary structure of this polysaccharide was demonstrated as follows: FA was composed of α-D-glucopyranose and β-D-glucopyranose with the α-(1→4) linkages in the main chain and the β-(1→6) linkages in side chain. FB and FC were composed of only α-D-glucopyranose with the (1→4)-linked-α-D-glucopyranosyl along the main chain. However, the elaborate structures of these three polysaccharides are still unclear and remain to be elucidated.

### 2.2. Effect of Three Fractions on PASM Cells Apoptosis under Hypoxia by Flow Cytometry

**Figure 3:**
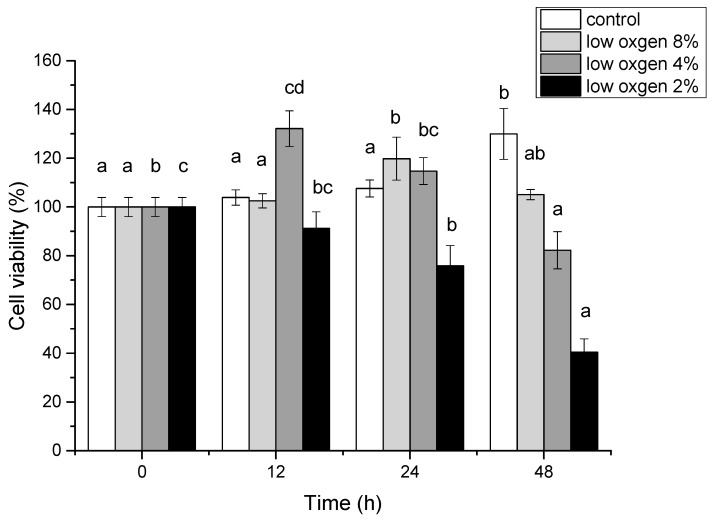
Effect of the designed hypoxia conditions at different times on cells survival.

PASM cell was used to evaluate the anti-hypoxia activities of FA in normal and hypoxia oxygen (2%, 4%, and 8% oxygen level) conditions by CCK-8 method. At the stress of hypoxia for 12 h, 24 h and 48 h, PASM cells showed a significant reduction of cell growth (Figure 3). If treated at 2% oxygen level for 24 h, PASM cells reduced in contrast to the control (normal oxygen level). The prolong of treatment time can greatly induce most PASM cells death. In combination with the above results, using 2% oxygen for treating 24 h was determined to be a favorable condition in the following study.

**Figure 4:**
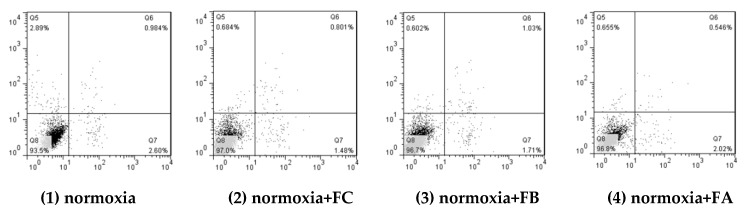

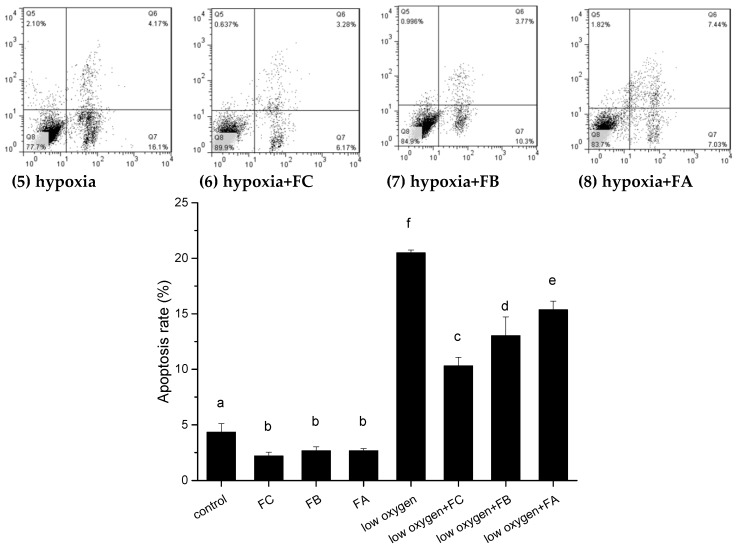
Hypoxia-induced apoptosis and the protective effect of three polysaccharide fractions after 24 h of treatment on PASM cells. Percentages reflect the total apoptotic cells (pre-apoptotic and apoptotic: quadrants two and four combined) pertreatment for means with different letters are significantly different from each other (*p* < 0.05).

After that, cells viability data were presented in Figure 4, employing flow cytometry. Compared to the normoxic group, hypoxia for 24 h resulted in marked increase of the apoptosis rates of PASM cells (*p* < 0.01). The apoptosis rate of PASM cell under hypoxia (2% oxygen) had increased to 21.42%, which was 5.60 times higher than the normoxia. Under hypoxia, the apoptosis rates of PASM cell treated with 250 µg/mL FA, FB and FC had significantly decreased 10.05%, 12.50%, and 15.06% respectively (*p* < 0.01) when compared to the hypoxia control group (21.42%). Under the normal oxygen condition, three polysaccharide fractions also can inhibit cells apoptosis. Among them, FC fraction showed the best anti-hypoxia effect under both the limited oxygen and normoxia.

Upon FA, FB and FC treatments, the cells appeared in late apoptosis and early apoptosis were significantly lower than that of the control (without polysaccharides under hypoxia). The active cells after polysacchairde fractions treatment were higher than that of the control group. Among them, FC had the best beneficial effect on inhibiting cell apoptosis under hypoxia. This means that the polysaccharide fractions from fruiting body of this mushroom demonstrated a capability to inhibit PASM cells apoptosis under hypoxia. As a consequence, the signaling pathways of different dose-inducing apoptosis need to be further explained.

### 2.3. Effect of Three Polysaccharide Fractions on Cell Compensation Responses to Acute Hypoxia

**Figure 5:**
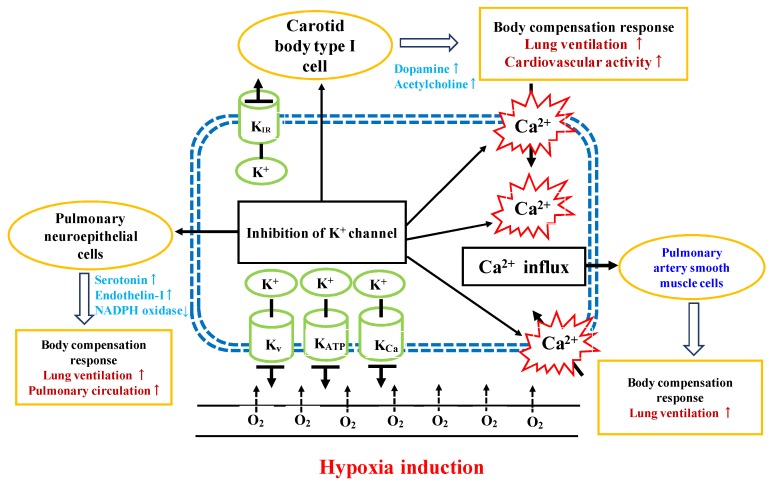
Postulated regulation pathway of oxygen-sensitive potassium channels on compensatory responses in acute hypoxia conditions.

Considering the molecular identity of the O_2_-sensor in PASMCs, the precise mechanisms that explain how hypoxic condition inhibit the K^+^ channels remain unclear, although several pathways have been proposed. Hypoxia may inhibit K^+^-channel activity by regulating one or more of the following: reactive oxygen species production, redox status and cell metabolism, and/or oxygen-sensitive molecules that are part of the channel or are closely associated to the channel protein [15]. Interestingly, none of these mechanisms are able to explain all of the data available, so it is likely that there is probably no single unique O_2_-sensing mechanism within the PASM cell. The postulated mechanism of hypoxia on PASM cells was provided in Figure 5. Though it has been known for many years that these stimuli increase cerebral blood flow, defining the mechanisms regulating these responses has been difficult. Upon cell compensation responses mechanism, we also investigated the six response items after three polysaccharide fractions were added to cell incubating system under hypoxia.

**Figure 6:**
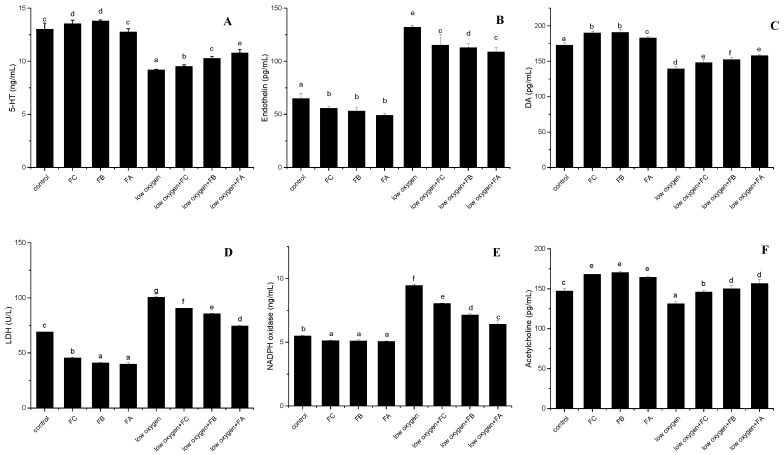
Effects of three polysaccharide fractions (**A**–**F**) on cell compensation responses under hypoxic stress.

5-hydroxytryptamine. The most well-known function of 5-hydroxytryptamine (5-HT) in the central nervous system (CNS) is neuromodulation, in processes such as memory, learning, mood, and the sleep-wake cycle; all of these are regulated by this biogenic amine through a wide family of receptors [16]. 5-HT is a strong vasoconstrictor and a smooth muscle contraction stimulator. To determine whether three kinds of polysaccharide fractions can regulate 5-HT production in peripheral tissues, 5-HT was determined in the culture medium using Griess reagent [16]. Treatment of PASM cells with FA, FB, FC (250 μg/mL) can obviously induce the secretion of 5-HT to some extent under hypoxia (Figure 6A). Nevertheless, in contrast to the normal oxygen condition, the hypoxia stress significantly reduced the production of 5-HT of PASM cells regardless of whether it was supplemented by three polysaccharide fractions. In particular, FA, FB, and FC fractions showed negligable difference for 5-HT production.

Endothelins. Endothelins are peptides with receptors and effects in many body organs. Endothelin constricts blood vessels and raises blood pressure [17,18]. As presented in Figure 6B, compared to the normoxic group, hypoxial treatment for 24 h resulted in a marked increase of the contents of endothelins in PASM cells. Treatment of PASM cells with FA, FB and FC (250 μg/mL) significantly reduced the secretion of endothelins (Figure 6B). Noticeably, three kinds of polysaccharide fractions showed insignificant differences under hypoxia. It is well known that the reduction of endothelins likely improved the response to hypoxia stress.

Dopamine. Dopamine (DA) is an important organic chemical in brain and body which is of the catecholamine and phenethylamine families. Its precursor is L-DOPAs which are synthesized in the brain and kidneys. To Synthesize DA, a carboxyl group is removed from one molecule of L-DOPA. Outside the central nervous system, DA functions primarily as a local chemical messenger. In blood vessels, it inhibits norepinephrine release and acts as a vasodilator [19]. Treatment of PASM cells with FA, FB and FC fractions significantly enhanced the secretion of DA to some extent under hypoxia (Figure 6C). It is well acknowledged that FA showed a beneficial effect on improving DA formation in PASM cells under hypoxia, inferring that the glucosidic linkages may be helpful for anti-hypoxia activities.

Lactate dehydrogenase. Lactate dehydrogenase (LDH) is an enzyme found in nearly all living cells. LDH catalyzes the conversion of lactate to pyruvic acid and back, as it converts NAD+ to NADH and back. LDH is expressed extensively in body tissues, such as blood cells and heart muscle. Owing to it being released during tissue damage, LDH is a marker of common injuries and diseases such as heart failure [20]. As shown in Figure 6D, compared to the normoxic group, hypoxial treatment for 24 h resulted in a major increase of the contents of LDH in PASM cells. Interestingly, treatments with FA, FB, FC for 24 h all showed significant decreases of LDH activity in PASM cells under hypoxia. While LDH activity is correlated to muscle fatigue, the production of lactate by means of LDH complex works as a system to delay the onset of muscle fatigue [21]. It is implied that polysaccharide fractions derived from the fruiting body of this mushroom possess a favorable effect on PASM cells survival under hypoxia.

NADPH oxidase. Under normal oxygen circumstances, NADPH oxidase is usually dormant, but during respiratory burst, it is activated and assembled in the membranes. The generation of superoxide is induced by the activation of NADPH oxidase and superoxide plays an important roles in plant signaling and animal immune response. Excessive ROS in vascular cells leads to various cardiovascular diseases including myocardial infarction, hypertension, ischemic stroke and atherosclerosis [22]. To investigate the effect of polysaccharide fractions on NADPH oxidase expression under hypoxia, the treatments of PASM cells with three fractions were conducted. Results demonstrated that hypoxial treatment can significantly enhance the expression of NADPH oxidase if compared to the normixal condition, inferring that PASM cells may accumulate more ROS in tissues than the normal condition (Figure 6E). As compared to the untreated cells, the treatments by FA, FB, FC lead to a significant decreasing effect on NADPH oxidase expression upon hypoxia, indicating that polysaccharide fractions may strengthen the compensatory responses in acute hypoxia conditions [23]. It is noteworthy that FA is favorable for regulating NADPH oxidase expression during limited oxygen circumstances.

Acetylcholine. Acetylcholine functions in both the central nervous system (CNS) and the peripheral nervous system (PNS). In the CNS, cholinergic projections from the basal forebrain to the cerebral cortex and hippocampus support the cognitive functions of those target areas. In the PNS, acetylcholine activates muscles and is a major neurotransmitter in the autonomic nervous system [24]. Figure 6F illustrates the effect of hypoxia treatment in combination with polysaccharide fractions on acetylcholine production in PASM cells. Compared to the normixal cultivation, hypoxia treatment significantly inhibited the production of acetylcholine in PASM cells. The supplementation of polysaccharide fractions can significantly improve the formation of acetylcholine in contrast to the untreated control in PASM cells. The effects of three polysacharide fractions for regulating acetylcholine were not statistically different. Overall, the purified polysaccharide compounds play a key role in dilating blood vessels and then improving body compensatory responses.

### 2.4. Effect of Three Polysaccharide Fractions on Cell Compensation Responses to Acute Hypoxia

Recently, the inhibition of voltagegated potassium (Kv) channels by hypoxia has been reported to play a role in the vascular constriction induced by hypoxia. By extending the repolarization period for calcium entry, the Kv channels inhibition may be involved in hypoxia-induced vasoconstriction. The four subtypes of potassium channels in vascular smooth muscle cells are Kv, ATP-sensitive K^+^, inward rectification and large conductance Ca^2+^-activated K^+^, among which Kv1.2, Kv1.5 and Kv2.1 are sensitive to hypoxia, and were found to contribute to hypoxic cerebral vasoconstriction [25]. At present, at least three different K^+^ channels have been found to exist in the inner mitochondrial membrane (mitoKv1.3, mitoKATP and mitoBKCa), and their pharmacological and electrophysiological properties have been investigated in a number of models including mitoplasts [26,27], proteosomes, planar lipid bilayers, and intact cells [28,29]. There is evidence that the modulation of activity of these channels could contribute to cellular protection against hypoxic injury [30], and numerous attempts have been made to decipher their exact role in mitochondrial and cellular pathophysiology. However, the molecular structure of these channels and the protective mechanism of them are still topics of discussion.

**Figure 7:**
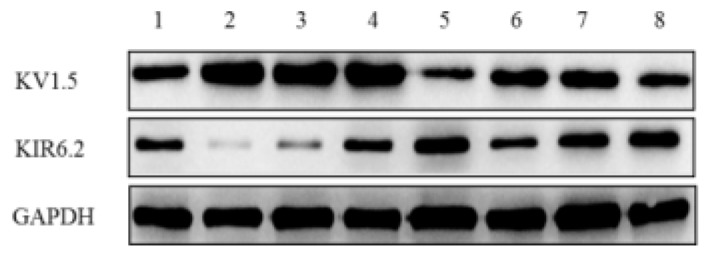

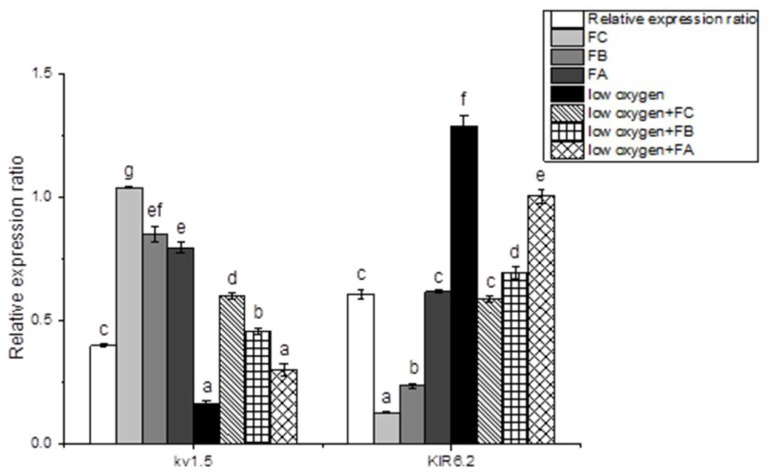
The effect of three fractions A, B and C on the expressions of KV1.5 and KIR6.2 under hypoxia with 2% oxygen content. Western blot analysis was implemented to present the expression of proteins. Densitometry analysis was employed to analyze the densities of brands. Data are presented after normalization by GAPDH. PASM cells were treated by FA, FB, FC (200 μM) for 24 h. All results were expressed in the form of means ± SD (n= 3). 1: control group under normal oxygen; 2: Fraction A group under normal oxygen; 3: Fraction B under normal oxygen; 4: Fraction C group under normal oxygen; 5: control group under hypoxia; 6: Fraction A group under hypoxia; 7: Fraction B group under hypoxia; 8: Fraction C group under hypoxia.

In terms of mechanisms underlying FA, FB and FC induced vasoconstriction under hypoxia, the Kv and K^+^ channels were involved, among which KV1.5 and KIR6.2 were sensitive to hypoxia [31,32]. To assess the mechanism of cell death in PASMCs induced by hypoxia, the two important proteins, KV1.5 and KIR6.2 related with the K^+^ channel were measured using the western blot technique. Figure 7 showed that the expression of KV1.5 has decreased under hypoxic stress, and the relative expression of KV1.5 protein in PASM cells treated with polysaccharide fractions has significantly increased under both normoxia and hypoxia, among which FA showed the best favorable effect on KV1.5 formation compared to the untreated group. The expression of KIR6.2 has increased under hypoxic condition in contrast to normoxia, while treatment with three fractions could decrease its expression to some extent. Specifically, FA greatly decreased the expression of KIR6.2 under hypoxic stress in PASM cells (*p* < 0.01), which can restore the damaged cells to normoxia state. It was found that the overexpression of KIR6.2 in mitochondria protects cells against hypoxic stress [27]. The present data of KIR6.2 showed the converse change against KIV1.5. The plasma membrane K^+^ channels activity is a vital determinant of membrane potential (Em). Previous findings revealed that the inhibition of K^+^ channels causes membrane depolarization, opens voltage-dependent calcium channels (VDCC), promotes Ca^2+^ influx, increases [Ca^2+^]cyt, and triggers PASM cells contraction [33]. Our results implied that an enhanced mitochondrial K^+^ uptake elicits cellular protection from hypoxic injury when supplemented with soluble polysaccharide fractions.

## 3. Materials and Methods

### 3.1. Materials and Reagents

The fruiting bodies of *A. bitorquis* (Quél.) Sacc. Chaidam mushroom collected from Qinghai Province, China, were provided by the Wild Plants Resources Institute of Qinghai Academy of Agriculture and Forestry Science (Xining, China). They were freeze-dried after removing any residual compost and were cut into pieces. Then, lyophilized mushrooms were grinded into powder. Reference monosaccharides (D-mannose, D-xylose, D-galactose, D-glucose, L-arabinose, L-rhamnose) were purchased from Sigma-Aldrich (St. Louis, MO, USA). Other chemicals used were of analytical grades.

### 3.2. Isolation and Fractionation of Aqueous Polysaccharides

The fruiting body powder of the tested mushroom was extracted with 50 times volume (g/mL) of distilled hot water (80 °C) for 3 h (3 times). The supernatant was collected and a certain amount of water was rotary evaporated at 60 °C under reduced pressure (0.02 kPa) [34]. The phenol-sulfuric acid method was employed for the detection of polysaccharide concentration. In brief, 1 mL polysaccharide solution (using glucose as the standard) was added into 0.5 mL 6% phenol and mixed with 5 mL concentrated sulfuric acid, then the absorbance at 490 nm was valued on a spectrophotometer [35]. The IPS concentration was 15.25 ± 0.13 g/100 g. After redissolving the freeze-dried crude IPSs in deionized water, the IPS solution was purified with a cellulose DEAE-52 column (2.6 cm × 40 cm) and a Sephacryl S-200 gel filtration column (2.0 cm × 60 cm) [33,34,35,36]. The IPS solution was eluted with double distilled water at a flow rate of 0.6 mL/min and detected by UV detector. A fraction collector was used to collect the dominating IPS fractions (5.0 mL/tube) according to the elution curves. The resulting pure polysaccharide fractions named FA, FB and FC were used in the subsequent chemical analyses and anti-hypoxia evaluation.

### 3.3. Molecular Weight Determination Analysis

The *Mw* of fractions FA, FB and FC was determined by high performance gel permeation chromatography (HPGPC). Waters 1525 HPLC system was employed for HPGPC. The column temperature was set at 35 °C. KH_2_PO_4_ solution (0.02 mol/L, 0.6 mL/min) was used for elucidation. Seven analytical standards of dextran (molecular weights: 5.0 × 10^3^–4.1 × 10^5^) were applied for the calibration curve. The molecular weight was calculated by referring to the standard dextran.

### 3.4. Chemical Analyses

Ultraviolet (UV) spectroscopy of FA, FB, FC fractions was performed on a Lambda UV spectrometer (Perkin Elmer, Norwalk, CT, USA). Samples were scanned from 200 nm to 400 nm. The monosaccharides composition of the polysaccharide samples was determined by GC. The procedure used was described by Yang et al. [37] with minor modifications. Monosaccharides composition was confirmed using the retention time of the chromatographic peak. The percentage of different monosaccharide was determined according to the area normalization method. GC was performed using a Shimadzu GC-2010 equipped with a capillary column of Rtx-1 (30 m × 0.25 mm × 0.25 µm) by the method of our previous work. The monosaccharide content can be calculated by the following Equation (1):

(1) $$Monosaccharide\;content\;\left(\%\right)=\frac{A1}{A2}\times \frac{V}{M}\times C$$

where *A*_1_ and *A*_2_ is peak area of are sample and standard peak areas, respectively; *V* is the sample volume constant volume (mL); *M* is weight the sample quality (g); *C* is the sample sugars concentration of mixed standard (mg/mL).

### 3.5. Nuclear Magnetic Resonance (NMR) Spectroscopy

Nuclear magnetic resonance (NMR) experiments (^1^H and ^13^C) were performed on a 600 MHz NMR spectrometer (DD2, Agilent) at 25 °C. For NMR spectroscopic analysis, IPS fractions (60 mg) were dissolved in 0.8 mL D_2_O and lyophilized three times. Before transferring into NMR microtubes, samples were finally dissolved in 0.6 mL of high quality D_2_O containing 0.1 μL acetone [38]. The chemical shifts of the purified fractions showed positive signals.

### 3.6. Cell Culture

Pulmonary artery smooth muscle cells (PASMCs) line was obtained from the Cell Bank of the Chinese Academy of Sciences (Shanghai, China). PASM cells were inoculated into a 96-well (4 × 10^3^ cell/well) plates and grown in a humidified incubator of 5% CO_2_ at 37 °C and cultured in DMEM medium containing 1% penicillin/streptomycin and 10% fetal bovine serum. The fresh medium was replaced every day. To harvest confluent cells, 0.25% trypsin-EDTA solution (Invitrogen, Shanghai, China) was used. Before treatment, PASM cells were allowed to attach on 96-well plates for 24 h. Then the purified fractions (FA, FB and FC) solution was added to the culture medium for treating for 24 h. Cells without any treatment (medium only) were used as the control in the following experiments.

### 3.7. Cell Viability Assay

The cell viability was determined using Cell Counting Kit (CCK8, Dojindo Laboratories, Kumamoto, Japan) following the instructions of the kit [39,40]. Herein, cell line was inoculated into 96-well plates (1 × 10^3^ cells per well). After incubating for 24 h, the media were replaced by fresh media with or without polysaccharide fractions. After culturing for 24 h, 48 h, and 72 h, the media were removed, and 100 μL medium containing 10 μL of CCK8 was added to each well followed by incubation for another 2 h. The absorbance at 450 nm was determined on a microplate reader (Thermo, Waltham, MA, USA) and each test was performed in triplicated experiments.

### 3.8. Detection of Cell Apoptosis

Apoptosis rate in PASM cells was determined by flow cytometry using the Annexin V-FITC conjugated apoptosis detection kit (BD Biosciences FACSCalibur, SP, USA). In brief, PASM cells (1 × 10^6^) were collected by centrifuging for 5 min at 1000 r/min and the cells were washed with pre-cooled PBS twice. Then the cells were re-suspended in binding buffer and 5 µL Annexin-V FITC were added in dark. After incubating for 10 min at room temperature in dark, 10 µL of propidium iodide (PI) was added immediately prior to analysis. The calibration was performed on cell quest pro software using flow cytometry (FACS Calibur, BD Bioscience). The cell viabilities were calculated according to the Equation (2):

(2) $$Cell\;viabilities\;\left(\%\right)=\frac{\mathrm{OD}\;\mathrm{hypoxia}\;\mathrm{control}\;\mathrm{or}\;\mathrm{OD}\;\mathrm{sample}}{\mathrm{OD}\;\mathrm{normal}\;\mathrm{control}}\times 100\%$$

### 3.9. Western Blot Analysis

PASM cells were harvested by scraping with RIPA buffer. Extracts were incubated for 2 h at 4 °C and obtained by centrifugation (13,000 rpm for 20 min at 4 °C). Protein concentrations were determined using the BCA assay Kit (Thermostat, Hercules, CA, USA), and whole-cell extracts were adjusted to same amount of total protein (20 μg). Samples were electrophoresed in 10% SDS-PAGE. Then proteins were transferred onto a PVDF membrane (Millipore Corporation, Billerica, MA, USA) at 300 mA for 90 min at 4 °C, and the membranes were incubated with 3% BSA (Sigma-Aldrich, St. Louis, MO, USA) in TBST to block nonspecific binding. Primary antibodies were incubated over night at 4 °C. We also washed five times with TBST (0.5% tween 20 in 1x TBS) the secondary antibodies conjugated horseradish peroxidase (HRP) (Santa Cruz, Dallas, Texas, USA) that was applied and incubated for 1 h at RT. After five times of washing with TBST (0.5% tween 20 in 1x TBS), the membrane followed by chemiluminescent detection using Immobilon Western substrate (Millipore Corporation, Billerica, MA, USA) with the ChemiDoc MP Imaging system (Bio-Rad Laboratories Inc., Hercules, CA, USA).

### 3.10. Determination of Enzymes Activity

LDH activity was measured in 20 mM Tris-HCl (pH 7.5), 0.15 M OD_sample_ NaCl, 300 μM NADH and 1 mM pyruvate, as described by Kaczor et al. [41] with some modification. The reaction was initiated by adding pyruvate. The lucigenin-enhanced chemiluminescence assay [39] was used to determine NADPH oxidase activity in cell homogenates. The activity was expressed as mean light units (ng)/mL of total protein.

### 3.11. ELISA Assay of Dopamine, 5-hydroxytryptamine, Endothelin and Acetylcholine

A microtiter plate format kit was used for the competitive dopamine (DA) ELISA assay. Dopamine is bound to the solid phase of the microtiter plate. Bounded DA and acylated DA from the sample compete for a certain number of antiserum binding sites [40]. Free antigen and free antigen-antiserum complexes are removed by washing after the system is in equilibrium. The solid phase DA bounded antibody is detected by anti-rabbit IgG/peroxidase. The substrate TMB/peroxidase reaction is monitored at 450 nm. The amount of the solid phase DA bounded antibody is inversely proportional to the sample DA concentration. Competitive inhibition enzyme immunoassay technique is employed for 5-HT determination [41]. Goat-anti-rabbit antibody has been pre-coated on the microtiter plate provided in this kit (CSB-E08364r). Standards or samples are added to the appropriate wells with antibody specific for 5-HT and horseradish peroxidase (HRP) conjugated 5-HT. The competitive inhibition reaction is initiated between the unlabeled 5-HT and HRP labeled 5-HT with the pre-coated 5-HT antibody. A substrate solution is added to the wells and the color develops in opposite to the amount of 5-HT in the sample (detection range: 0.8 ng/mL-160 ng/mL). After stopping the color development, the intensity of color is measured at 450 nm. The competitive inhibition enzyme immunoassay technique is employed for Endothelin (EDN) assay. The microplate has been pre-coated with a monoclonal antibody (MBS2024979) specific to endothelin 1 (EDN1). A competitive inhibition reaction is initiated between biotin labeled EDN1 and unlabeled EDN1 (Standards or samples) with the EDN1 antibody. After washing off the unbound conjugate, HRP conjugated avidin is added and incubated. The amount of bound HRP conjugate is inversely proportional to the concentration of EDN1 in the sample. Thus, the color intensity at 450 nm developed by adding substrate solution is also inversely proportional to the EDN1concentration. Acetylcholine assay kit (KA1624, Abnova Co., Taiwan) was used for acetylcholine determination. In brief, acetylcholine is hydrolyzed by acetylcholinesterase to choline which is oxidized by choline oxidase to betaine and H_2_O_2_. The resulting H_2_O_2_ reacts with a specific dye to form a pink colored product [42]. The color intensity at 570 nm or fluorescence intensity (530/585 nm) directly reflected the acetylcholine concentration in the tested samples. Samples were analyzed in triplicates.

### 3.12. Statistical Analysis

Data were presented as means ± standard deviations for three replicates for each sample. The data were statistically analyzed with the SPSS statistical software (SPSS Inc, USA). A probability of *p* < 0.05 was considered as statistical significance.

## 4. Conclusions

In summary, the aqueous polysaccharide fractions isolated from the fruiting body of *A. bitorquis* (Quél.) Sacc. Chaidam mushroom possess significant anti-hypoxia potential. Polysaccharide with low molecule weight (FC) showed good performance of againsthypoxia damage through decreasing LDH and NADPH oxidase expression, in combination with the increased formation of EDH, 5-HT and acetylcholine under hypoxia in PASM cell. Moreover, FC fraction demonstrated anti-hypoxia capacity by inducing KV1.5 expression and conversely inhibiting KIR6.2 expression. The findings of this work signify the potential of intracellular polysaccharides from this special underground mushroom as a promising source of natural anti-hypoxiacompounds. However, further research is needed on the elaborate structure of three polysaccharide fractions. The quantitative structure–activity relationship (QSAR) of these polysaccharides as well as their biological effects at molecular levels will be the focus of future research.
